# The Effect of Probabilistic Context on Implicit Temporal Expectations in Down Syndrome

**DOI:** 10.3389/fpsyg.2020.00369

**Published:** 2020-03-06

**Authors:** Giovanni Mento, Gaia Scerif, Umberto Granziol, Malida Franzoi, Silvia Lanfranchi

**Affiliations:** ^1^Department of General Psychology, University of Padova, Padova, Italy; ^2^Padua Neuroscience Center (PNC), University of Padova, Padova, Italy; ^3^Department of Experimental Psychology, University of Oxford, Oxford, United Kingdom; ^4^Scientific Institute, IRCCS “E. Medea”, Association “La Nostra Famiglia”, Treviso, Italy; ^5^Department of Developmental Psychology and Socialization, University of Padova, Padova, Italy

**Keywords:** down syndrome, proactive motor control, temporal expectations, local-global processing, dynamic temporal prediction task

## Abstract

One of the most important sources of predictability that human beings can exploit to create an internal representation of the external environment is the ability to implicitly build up subjective statistics of events’ temporal structure and, consequently, use this knowledge to prepare for future actions. Stimulus expectancy can be subjectively shaped by hierarchically nested sources of prediction, capitalizing on either local or global probabilistic rules. In order to better understand the nature of local-global proactive motor control in Down Syndrome, in the present study a group of participants with Down Syndrome (DS group; *n* = 28; mean age 29.5 ± 13 years; range 10–54) and a group of typically developing participants matched by either gender or mental age (TD-MA group; *n* = 28; 5.6 ± 1 years; range 4–8) were administered a novel motor preparation task, defined as the Dynamic Temporal Prediction (DTP) task. In the DTP, the temporal preparation to imperative stimuli is implicitly shaped by the local increase of expectancy. This is manipulated trial-by-trial as a function of the preparatory foreperiod interval (Stimulus-Onset Asynchrony or SOA). In addition, temporal preparation can be also implicitly adjusted as a function of global predictive context, so that a block-wise SOA-distribution bias toward a given preparatory interval might determine a high-order source of expectancy, with functional consequences on proactive motor control adjustment. Results showed that in both groups motor preparation was biased by temporal expectancy when this was locally manipulated within-trials. By contrast, only the TD-MA group was sensitive to global rule changes: only in this cohort was behavioral performance overall impacted by the SOA probabilistic distribution manipulated between-blocks. The evidence of a local-global dissociation in DS suggests that the use of flexible cognitive mechanisms to implicitly extract high-order probabilistic rules in order to build-up an internal model of the temporal properties of events is disrupted in this developmental disorder. Moreover, since the content of the information to be processed in the DTP task was neither verbal nor spatial, we suggest that atypical global processing in Down Syndrome is a domain-general rather than specific aspect characterizing the cognitive profile of this population.

## Introduction

Down syndrome (DS), one of the most common genetic syndromes, is caused by full or partial trisomy of chromosome 21, in particular Trisomy 21, which is the most common karyotype accounting for 95% of cases. DS affects about 1 in 1000 newborns (McGrother and Marshall, 2008). A core feature of most of people with DS is that they show mild to severe levels of intellectual impairment together with a wide range of associated physical, medical and neuropsychological deficits in several cognitive domains. More specifically, a consistent finding documented in the literature is that the neuropsychological profile of children with DS is characterized by some impaired domains (i.e., verbal abilities) in spite of other domains being relatively preserved (i.e., visuo-spatial skills). According to a neuroconstructive account, the domain-specific dissociations in the verbal and visuo-spatial cognitive functions observed in this syndrome might be better understood as the end-state of a process of atypical modularization emerging across development rather than as the starting point neuropsychological characteristic of DS (Karmiloff-Smith, 1998). Consistent with this account, previous studies have highlighted atypicalities in Executive Function (EF) as domain-general features at the basis of the domain-specific impairment described in the DS. Specifically, working memory, inhibition and flexibility/set-shifting have consistently shown to be weaker relative to typically developing children matched on mental age or children with other forms of intellectual disability (Lanfranchi et al., 2004, 2010; Lee et al., 2011; Borella et al., 2013; Carney et al., 2013; Costanzo et al., 2013; Daunhauer et al., 2017). In addition to EF difficulties, other researchers have emphasized differences in visual selective spatial attention (Cornish et al., 2007; Scerif and Steele, 2011; Breckenridge et al., 2013; Carney et al., 2013) and in visual and auditory sustained attention (Brown et al., 2003; Atkinson and Braddick, 2011) as possible early dysfunctional hallmarks constraining the development of domain-specific cognitive skills in DS.

In parallel with what was shown for the spatial domain, more recently it has been reported that the ability to use temporal information to implement attentional control and optimize behavior may constitute another domain-general property of the human cognitive system. In fact, the ability to selectively allocate attention in time (i.e., temporal orienting) plays an essential role in the proactive regulation of human behavior (Nobre, 2001). Specifically, the ability to use external or internal environmental cues to establish temporal expectancy toward upcoming events represents an important gating mechanism that enables us to prioritize relevant stimuli and to process them faster and better (Correa, 2010). While these mechanisms have been widely investigated in adults (Coull et al., 2000; Correa et al., 2004, 2006; Coull, 2010; Mento et al., 2015; Mento, 2017), only a handful of studies have addressed the developmental trajectory of temporal orienting mechanisms. In one of the earliest studies on this topic (Mento and Tarantino, 2015), we used a cued reaction time task to provide behavioral evidence that voluntary (top-down) and automatic (bottom-up) mechanisms at the basis of temporal orienting follow a stable developmental trajectory after 6 years of age, although the ability to make a combined use of them emerges after 8 years of age (but see Johnson et al., 2015). Neuroimaging data further suggests that 8–12-year-old children engage adult-like neural mechanisms to orient attention in time either voluntarily or automatically (Mento and Vallesi, 2016; Mento et al., 2018). In spite of this promising research line on typically developing children, temporal attention has slipped out of the research agenda in the study of atypical development. Yet, there is consensus that a failure in using temporal information to generate predictive behavior toward future events may be a hallmark common to several neurodevelopmental disorders (Brenner, 2012).

In a recent study (Mento et al., 2019), for the first time we extended the investigation of temporal orienting toward an understanding of the mechanisms underlying this function in atypical development. Specifically, a group of children with DS was compared with either chronological or mental age matched controls while performing the same temporal orienting task already used with typically developing children in Mento and Tarantino (2015). The results showed that the overall behavioral performance of participants with DS was similar to that of typically developing children with equivalent mental age both in terms of response speed and accuracy. In spite of this, while automatic temporal orienting mechanisms instantiated by temporal regularities (i.e., foreperiod intervals) seemed to be well established and operating in both typically developing children and individuals with DS, only the first were able to use voluntary temporal attention, taking advantage from the temporal information explicitly provided by symbolic cues to speed up their performance. In other words, the ability to implicitly represent and process temporal information to implement proactive motor control was preserved in DS, while the ability to represent and use such information explicitly was disrupted in this population. This finding is consistent with previous suggestions that the implementation of strategic processes engaged in top-down attentional control presents an additional challenge for people with DS (Lanfranchi et al., 2004, 2010). At a first glance, these findings seem to suggest a tout-court dissociation between explicit and implicit attentional mechanisms in DS. However, it is not entirely clear whether the nature of implicit processes in atypical development is completely comparable to what is known about typical development. Although previous studies have suggested that implicit learning is a developmentally stable and inflexible mechanism (Meulemans et al., 1998; Vinter and Perruchet, 2010; Amso and Davidow, 2012), evidence coming from infant studies indicates that this cognitive function might be a flexible rather than static process, able to adapt to different experiential contexts. An emblematic example is constituted by the so-called *rule learning*, that is, the ability to automatically abstract important information on the simple basis of the statistical properties of sensory events (e.g., syllables) and, consequently, to translate these rules to different domains (e.g., faces). In this regard, it has been reported that rule learning stabilizes within the first year of life in typically developing infants (Johnson et al., 2009). It is therefore plausible to suggest that, by school age, implicit learning extracts dynamic and flexible characteristics that in turn allow information to be acquired in automatic mode and across several environmental contexts. In other words, to facilitate the acquisition of new knowledge of the world, implicit learning should be a flexible and adaptive mechanism.

Following up this hypothesis, in a recent study (Mento and Granziol, under revision) we tried to provide empirical support for the hypothesis that implicit mechanisms underlying proactive motor control have a flexible nature. Namely, in this study we used a new, child-friendly reaction time task purposely designed to investigate how local (within-trial expectancy bias) and global (between-block expectancy bias) predictions interplay to generate temporal expectancy and consequently shape proactive motor control in young (5–6-year-old), middle (7–8 year old) and old (9–10 old) typically developing children. Interestingly, we found that while local temporal prediction showed stable developmental trajectories, the ability to use a block-wise global rule to proactively adjust motor control in terms of both accuracy and speed becomes stable after the age of seven. On the one hand, these findings support the view that the implicit learning is an early emerging mechanism that nevertheless goes through developmental changes during childhood. On the other hand, they suggest that flexibility of implicit learning may constitute a necessary prerequisite for mastering complex domains that are explicitly represented.

Coming back to DS, one of the characteristic aspects of this genetic disorder is the difficulty manifested in cognitive processes that imply the ability to represent and explicitly manipulate information. In contrast, implicit processing seems to be preserved (e.g., Mento et al., 2019). However, current studies have mostly used experimental tasks that imply a static, rather than flexible use of implicit cognitive resources. It is therefore not clear whether in a situation that requires cognitive flexibility in the implicit adaptation to a variable environmental context, individuals with DS have typical or atypical developmental characteristics. In line with what has been described in the literature for explicit flexibility (D’Souza et al., 2016), one might expect that, even in the case in which explicit and intentional representation and/or manipulation of information is not required, individuals with DS may have implicit learning difficulties within of a dynamic and variable context implying local – global rule shifts. This hypothesis is in line with the neuroconstructivist account, which postulates that an impairment of early and domain-general ability (such as implicit learning) could result in a cascade of atypical outcomes. The main aim of the present research is to investigate how local - global predictive contexts can implicitly influence the ability to orient attention over time and consequently modulate motor performance in DS. In line with previous studies (Mento et al., 2019), we expected individuals with DS to be able to implement a form of implicit temporal expectation, therefore being sensitive to simple predictive rules (local bias). In line with the difficulties of cognitive flexibility in explicit tasks, however, we expect that implicit temporal expectation may nonetheless be inflexible. In other words, we hypothesized that, compared to a control group of same mental age, individuals with DS may have difficulties in implicitly adapting their motor performance to global predictive rules in spite of preserved local prediction.

## Materials and Methods

### Participants

Twenty-eight participants with DS (mean age 29.5 ± 13 years; range 10–54; 14 females) were initially enrolled from different local associations of the North-East of Italy and compared with a group of twenty-eight mental age-matched participants with typical development (TD-MA; 5.6 ± 1 years; range 4–8; 14 females) recruited from preschools and primary Schools in the North-East of Italy. In both DS and TD-MA groups non-verbal abilities were assessed by means of the Raven’s Colored Progressive Matrices (CPM) test (Raven et al., 1990). The raw score obtained through the Raven’s PCM test was used to estimate mental age of both groups. For this purpose, Italian normative data (Belacchi et al., 2008) were used. The demographic characteristics of the three groups are reported in Table 1.

**TABLE 1:** Main demographic characteristics of the study’s participants.

	**Age (Years)**	**Mental Age**	**Gender**		
				
**Group**	**Mean ± SD (range)**	**Mean ± SD (range)**	**Female**	**Male**	***n***
DS	29.5 ± 13 (10–54)	5.57 ± 1 (3.5–8.5)	14	14	28
TD-MA	5.6 ± 1 (4–8)	5.81 ± 1(3.5–8.5)	14	14	28

*SD, standard deviation.*

Furthermore, parents of participants were asked to fill in the SDAG (Scala di Disattenzione e Iperattività – Genitori, or the inattention and hyperactivity scale for parents (Cornoldi et al., 1996). This is an Italian parent report measure designed to assess the presence of inattentive (subscale SDAG-1) or hyperactive (subscale SDAG-2) symptoms in children. Children scoring above the critical cut-off for the presence of significant inattentive or hyperactive/impulsive behavior were excluded from the study. Children reported as having neurological or psychiatric conditions were also excluded. All children’s parents signed a written consent form. All experimental procedures were approved by the Ethics Committee of the School of Psychology at the University of Padua (protocol n° 2536) and were conducted according to the principles expressed in the Declaration of Helsinki.

### Experimental Procedure

All participants contributed to the study individually in a quiet room. Stimuli were presented on a laptop with a 17-inch monitor at a resolution of 1,280 × 1,024 pixels. Participants were seated comfortably in a chair at a viewing distance of around 60 cm from the monitor. All participants performed the Dynamic Temporal Prediction (DTP; Mento and Granziol, under revision) task. The DTP has been adapted from a previous task in our laboratory (Mento and Tarantino, 2015) to investigate how children adapt proactive motor control in relation to dynamic changes in the local and global predictive rules across the task.

### Trial Structure

Each trial began with the display of a warning visual stimulus (S1), followed by the presentation of an imperative visual stimulus (S2) that stayed on the screen for a maximum of 3,000 ms. S1 consisted of a picture of a black camera lens surrounded by a circle (total size of the stimulus: 840 × 840 pixels, 144 dpi, 10.62° × 10.54° of visual angle). S2 consisted of a picture of a cartoon character, which was displayed centrally within the camera lens. The inter-trial-interval was randomly manipulated between 600 and 1,500 ms. The task consisted of speeded target detection; participants were required to press the space bar with the index finger of the dominant hand as quickly as possible at target occurrence. To encourage good performance in the participants, they were given the following instruction:

Hi! This is the BARBAPAPA family! Here is Barbadad, Barbamama, and their seven children. The barpapapas are playing hide and seek in the woods. Your job is to take a photo of them as soon as they appear in view of your camera. You can take a photo by pressing the space bar. Find them all! But take care—if you press the bar too soon or too late, they will run away!

### Local Predictive Context

**FIGURE 1:**
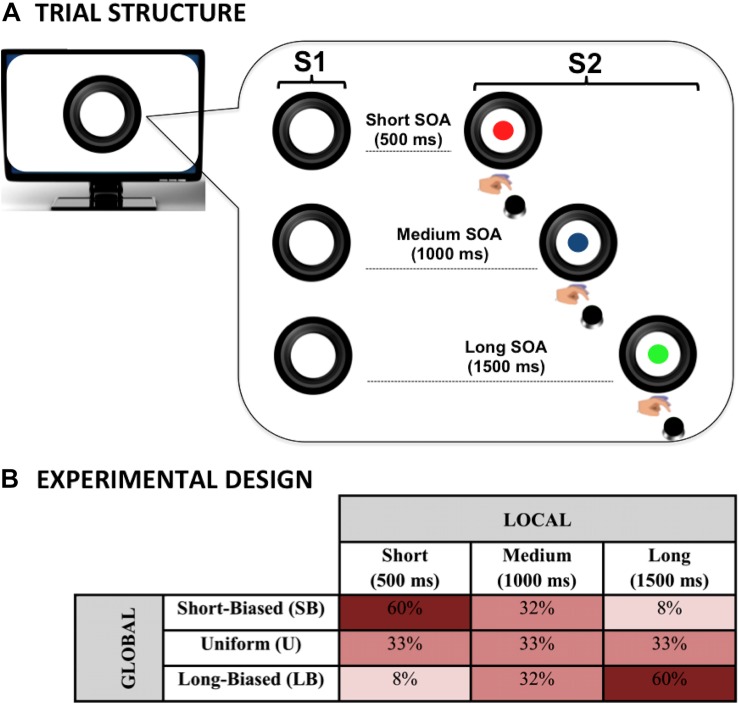
Dynamic temporal prediction (DTP) task. The DTP task was purposely designed to investigate the effect of both local and global predictive rules on implicit temporal preparation. The circle (S1) warned children on the presentation of the imperative S2 stimulus (a cartoon character; here represented with colored disks for illustrative purposes due to copyright restriction). Participants had to make speeded reaction times at S2 onset by pressing a button on the keyboard. The effect of local prediction was assessed by manipulating S1–S2 stimulus onset asynchrony (SOA) within each experimental block **(A)**. The effect of global prediction was assessed by manipulating the between-block, *a priori* relative SOA distribution to create three probabilistic distributions in which the SOAs were equally distributed (uniform) or skewed toward the short (short-biased) or long (long-biased) SOA **(B)**.

To investigate the effect of the local predictive context on behavioral performance, the S1–S2 stimulus-onset-asynchrony (SOA) was varied trial by trial within each experimental block so that three possible fixed intervals were created. These included a short (500 ms), a medium (1,000 ms), or a long (1,500 ms) SOA. This manipulation, illustrated in Figure 1A, was intended to introduce three levels of temporal preparation to S2 onset in each block. Specifically, this manipulation allowed us to investigate local prediction as the effect of the stimulus hazard rate on task performance. Indeed, the use of an S1–S2 SOA variable is expected to dynamically bias subjective temporal expectancy (Woodrow, 1914; Karlin, 1958; Niemi and Näätänen, 1981; Luce, 1986; Nobre et al., 2007; Los, 2010). Specifically, in line with previous literature (see Los, 2010 for a review), we expected participants to be fastest at detecting the targets occurring at the longest SOA and slowest at those appearing at the shortest SOA.

### Global Predictive Context

As illustrated in Figure 1B, to assess the effect of the global changes in the predictive context, different probability distributions per each SOA interval were introduced and manipulated block-wise, as described below.

#### Uniform (U) Distribution Block

In this condition, a rectangular distribution of the three SOAs was used. That is, the frequency of each SOA in a block was kept constant. This type of distribution is the most classic probabilistic distribution employed in both the adult (Los, 2010; Vallesi, 2010; Mento et al., 2015; Mento, 2017) and the developmental SOA literature (Vallesi and Shallice, 2007; Johnson et al., 2015; Mento and Tarantino, 2015; Mento and Vallesi, 2016).

The use of an *a priori* uniform distribution has long been described to translate into a biased *a posteriori* temporal preparation. Indeed, as time goes by, the conditional probability of S2 onset increases exponentially in virtue of the fact that it has not occurred yet (Luce, 1986; Nobre et al., 2007). As a consequence, motor preparedness will be lowest at the shortest SOA and highest at the longest SOA.

#### Short-Biased (SB) Distribution Block

In this case, an *a priori* biased distribution toward the short SOA was delivered. In particular, the relative percentage was 60, 32, and 8% for the short, medium, and long SOA, respectively. This kind of distribution, also known in the literature as *non-aging* distribution (Trillenberg et al., 2000; Los et al., 2017), is purposely intended to counterbalance the hazard-based increment of temporal expectancy as a function of SOA length.

#### Long-Biased (LB) Distribution Block

In this block, a distribution mirroring the one used in the SB block was created. Specifically, the relative percentage was 8, 32, and 60% for the short, medium, and long SOA, respectively. This kind of distribution, also known in the literature as aging distribution (Trillenberg et al., 2000; Los et al., 2017), is purposely intended to exacerbate the hazard-based increment of temporal expectancy as a function of SOA length.

### Experimental Design

The experimental manipulations yielded a factorial design in which either the SOA (short versus medium versus long) and the block type (SB versus U versus LB) factors were orthogonally contrasted to investigate the effect of local and global predictive context, respectively. A total of three experimental blocks per SOA distribution were delivered, for a total of 9 blocks. Each block included 30 trials, for a total of 270 trials administered to each participant. The total length of the experiment was about 15 min. To avoid participants inferring the change in the global probability distribution, no pauses were introduced between blocks. Instead, a blank slide was inserted in the middle of each block to allow children to rest. It is important to note that participants were not told about the presence of between-block different probabilistic distributions to ensure they did not know about the global rule changes. In this way, we were able to investigate the presence of group differences in relation to the ability to adjust behavioral performance implicitly in terms of both speed (RT) and accuracy (percentages of not-anticipated responses) as a function of either local or global predictive rules. All blocks were matched for sensorimotor requirements, as the visual stimuli and the required response were always the same across the experiment. The only differences were related to the changes in the predictive context experienced through the task. Moreover, block-type order was counterbalanced between subjects. This ensured that spurious effects due to introducing either local or global fixed predictive contexts did not bias the performance. Before starting the experimental session, participants were presented with a block of 20 training trials for each condition to ensure they understood task instructions. During training, all participants received feedback every trial according to their performance. Specifically, a neutral yellow smile was displayed in cases in which either anticipatory (before target onset) or premature (<150 ms before target onset) responses were provided. A yellow smile was displayed if the RT was between 1,000 and 1,500 ms from target onset. Finally, a green smile was displayed if the RT was between 150 and 1000 ms. E-prime 2 software (Psychology Software Tools, Pittsburgh, United States) was used to create and administer the experiment.

### Data Analysis

Both mean accuracy and RT to targets were collected and analyzed separately for both groups. Specifically, in order to obtain a preliminary, general measure of the ability to accomplish the task, mean accuracy was calculated for each group as the mean of correct (i.e., not anticipated) responses across all experimental conditions. Only responses between 150 ms and 1,500 ms from target onset were considered correct and included in the analyses. According to our previous study investigating motor preparation in participants with DS (Mento et al., 2019), only participants showing mean performance exceeding an *a priori* cutoff value of >65% were entered in the models on RTs. This was done to rule out the possibility that any RT effects or their comparisons across groups may be biased by spurious variables, including poor understanding of task instruction or generalized difficulties in maintaining attentional set along the whole task. This also allowed us to improve the statistical reliability of RT analysis because it only included those participants who exhibited a sufficient number of correct trials per experimental condition.

Generalized linear mixed effect models (GLMMs) were tested on both mean accuracy and RTs. Group (i.e., DS vs. TD-MA), the SOA length within-block (i.e., short, medium and long) and the block-type (SB_d_ vs. U_d_ vs. LB_d_) were set as fixed factors, the models’ intercept as random factor (i.e., random intercept models) and children as the clustering variable. Interactions of Group with both SOA and Block were tested as well. Cohen’s *d* according to the method explained by Westfall et al. (2014) were computed for each effect (i.e., main or multiple comparisons). All the statistical analyses were computed using R statistical software (R Core Team, 2018), and using the *lme4* package (Bates et al., 2015) to test the GLMMs. Given the exploratory nature of this study, multiple comparisons were computed only for the statistically significant main effects, by using the *emmeans* (Lenth, 2018) package; in this case, the *p*-value were adjusted with a False Discovery Rate correction (FDR; (Benjamini and Yekutieli, 2001). The *p*-values of the GLMMs were obtained by means of the *car* package (Fox and Weisberg, 2018). The results of the GLMMs are presented separately.

## Results

### Accuracy

The mean accuracy scores per group and condition are reported in Table 2. The statistical results for task accuracy are summarized in Table 3.

**TABLE 2:** Mean accuracy.

	**SB_S_**	**SB_M_**	**SB_L_**	**U_S_**	**U_M_**	**U_L_**	**LB_S_**	**LB_M_**	**LB_L_**
DS	94.3(8)	89.8(13)	91.1(8)	94.4(8)	90.3(13)	86.1(19)	94.6(12)	92.5(10)	88.6(14)
TD-MA	96.7(4)	89.4(9)	86.3(15)	98.1(2)	93.1(2)	86.6(7)	98.2(5)	94.3(7)	89.7(7)

*Mean and standard deviation (in parentheses) measures of accuracy (percentage of correct responses) for participants with Down Syndrome (DS) and typically developing participant matched by mental age (TD-MA) for each experimental condition. Specifically, the leftward columns report data for the short-biased (SB) blocks relatively to the short, medium and long SOA (SB_*S*_, SB_*M*_ and SB_*L*_, respectively). The central columns report data for the uniform (U) blocks relatively to the short, medium and long SOA (U_*S*_, U_*M*_ and U_*L*_, respectively). The rightward columns report data for the long-biased (LB) blocks relatively to the short, medium and long SOA (LB_*S*_, LB_*M*_ and LB_*L*_, respectively).*

**TABLE 3:** Main results of the generalized linear mixed-effect model on mean accuracy.

**Predictors**	***X*^2^**	**Df**	***p*-value**
Group	0.49	1	0.48
SOA	106.41	2	<0.0001
Block	5.41	2	0.07
Group × SOA	5.98	2	0.05
Group × Block	6.03	2	0.04

*Main results of GLMM on mean accuracy. Statistics refer to Wald chi-square test, with degrees of freedom calculated according to Satterthwaite approximation.*

**FIGURE 2:**
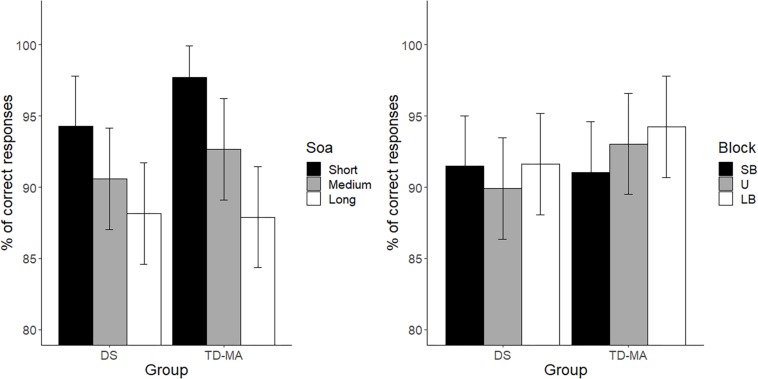
Mean task accuracy. Age cluster interacts with foreperiod (**left panel**) and distribution (**right panel**). LB, long-biased; SB short-biased; SOA, stimulus-onset-asynchrony; U, uniform. Black bars refer to confidence intervals.

There were no significant between-group differences in terms of mean accuracy (percentages of anticipated responses), confirming that the task was equally difficult for participants with DS and TD-MA children. Nevertheless, accuracy was affected by SOA, because participants were overall more accurate at detecting the trials with short as compared to those with both medium (*t*_(__424__)_ = 5.68, *p* < 0.001, *d* = 0.39) and long SOA (*t*_(__424_^1^) = 10.29, *p* < 0.001, *d* = 0.72). We also observed higher accuracy in medium than long SOA trials (*t*_(__424__)_ = 4.61, *p* < 0.001, *d* = 0.32). Although the Group × SOA interaction did not reach statistical significance the data show a general tendency of the DS group to make more anticipation errors. In fact, as shown in Figure 2 (left panel) the accuracy difference between the short and long SOA trials was bigger in the TD-MA group (local delta effect = 9.84%; *t*_(__424__)_ = 8.99, *p* < 0.001, *d* = 0.89) than in participants with DS (local delta effect = 6.1%; *t*_(__424__)_ = 5.56, *p* < 0.001, *d* = 0.55).

No significant main effect of Block emerged, suggesting that accuracy was generally not affected by the global distribution properties of the single intervals. Most remarkably, we found a significant interaction between the factor Block and Group. As shown in Figure 2 (right panel), only in the TD-MA group was performance significantly affected by the global rule changes, with lower accuracy within SB than the LB blocks (*t*_(__424__)_ = 2.92, *p* = 0.01, *d* = 0.29). In contrast, DS children displayed similar accuracy (*t*_(__424__)_ = 0.19, *p* = 0.98, *d* = 0.01) in all blocks.

### Reaction Times

The mean accuracy scores per group and condition are reported in Table 4. The statistical results for task response speed are summarized in Table 5.

**TABLE 4:** Main results of the generalized linear mixed-effect model on mean reaction times.

**Predictors**	***X*^2^**	**Df**	***p*-value**
Group	1.44	1	0.23
SOA	46.32	2	<0.0001
Block	3.63	2	0.16
Group × SOA	0.09	2	0.95
Group × Block	9.18	2	0.01

*Main results of GLMM on mean reaction tie s (RTs). Statistics refer to Wald chi-square test, with degrees of freedom calculated according to Satterthwaite approximation.*

**TABLE 5:** Mean reaction times.

	**SB_S_**	**SB_M_**	**SB_L_**	**U_S_**	**U_M_**	**U_L_**	**LB_S_**	**LB_M_**	**LB_L_**
DS	844(241)	723(193)	765(318)	846(292)	742(275)	739(247)	847(307)	792(10)	703(225)
TD-MA	714(157)	658(176)	647(193)	853(189)	716(191)	656(155)	787(269)	726(192)	691(157)

*Mean and standard deviation (in parentheses) measures of reaction times for participants with Down Syndrome (DS) and typically developing participant matched by mental age (TD-MA) for each experimental condition. Specifically, the leftward columns report data for the short-biased (SB) blocks relatively to the short, medium and long SOA (SB_*S*_, SB_*M*_ and SB_*L*_, respectively). The central columns report data for the uniform (U) blocks relatively to the short, medium and long SOA (U_*S*_, U_*M*_, and U_*L*_, respectively). The rightward columns report data for the long-biased (LB) blocks relatively to the short, medium and long SOA (LB_*S*_, LB_*M*_ and LB_*L*_, respectively).*

**FIGURE 3:**
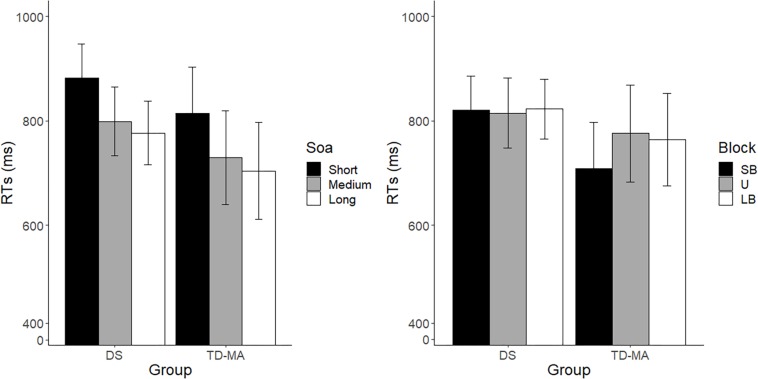
Mean reaction times. Age cluster interacts with SOA (**left panel**) and block (**right panel**) on RT. LB, long-biased; SB, short-biased; SOA, stimulus-onset-asynchrony; U, uniform; RT, reaction time. Black bars refer to confidence intervals.

In line with what was observed for accuracy, performance was comparable between participants with DS and TD-MA children in terms of response speed, as confirmed by the absence of a main Group effect on mean RTs. In spite of this, we observed a robust effect of SOA. As shown in Figure 3 (left panel), response speed was overall lower as the SOA increased, confirming that participants were able to adapt their motor preparation on the basis of the probability of S2 onset, which was lowest at the shortest SOA and highest at the longest SOA. More specifically, pairwise comparisons confirmed that participants were overall faster in the long than the short SOA trials (*z* = −6.75, *p* < 0.001, *d* = 1.16) as well as in the medium as compared to short SOA ones (*z* = −4.881, *p* < 0.001, *d* = 0.91). No significant differences were observed when comparing RTs in long *vs.* medium SOA trials (*z* = −1.618, *p* = 0.24, *d* = 0.25). Noticeably, the SOA effect was constant among groups, as confirmed by the non-significant interaction. Indeed, as displayed in Figure 3 (left panel), the difference between the mean response speed in short *vs.* long SOA trials was similar among the DS (local delta effect = 103.69 ms; *z* = 5.53, *p* < 0.001, *d* = 1.13) and TD-MA (local delta effect = 109.91 ms; *z* = 5.32, *p* < 0.001, *d* = 1.19) group.

No main effect of Block emerged, although this factor interacted with Group. Indeed, as the task became more pressing (SB block), TD-MA children showed faster RTs compared to LB blocks (global delta effect = 55 ms, *z* = 0.84, *p* = 0.13, *d* = 0.6). By contrast, participants with DS did not show any significant between-block difference in response speed (global delta effect: 2.87 ms, *z* = 0.17, *p* = 0.98, *d* = 0.03), as shown in Figure 3 (right panel). In other words, TD-MA children succeeded to implicitly adapt their behavioral performance to task demands, becoming faster when the task became more pressing. However, participants with DS failed to show any adjustment of response speed as a function of task difficulty. The lack of speed adaptation to the global temporal pattern of the task in participants with DS did not depend on their demographic characteristics, as shown by the absence of significant correlations between delta global effect and chronological (*r* = −0.13; *p* = 0.48) or mental (*r* = −0.24; *p* = 0.22) age.

## Discussion

In the present study, we investigated how the ability to generate implicit temporal expectancy on the basis of local and global predictive rules may affect proactive motor control in individuals with DS compared to mental-age matched typically developing children.

To this purpose, we used a simple stimulus detection task, known as Dynamic Temporal Prediction task (DTP; Mento and Granziol, under review), in which all participants were asked to produce speeded motor responses to warned imperative stimuli. The preparatory interval (SOA) between the warning and the imperative stimulus was manipulated within trials to generate temporal expectancy on the basis of local probabilistic rules. In addition, we introduced a higher-order (global) predictive rule by introducing three types of blocks with different SOA probabilities, leading to a U (same probability per each SOA), an SB (higher probability of short SOA), and an LB (higher probability of long SOA) distribution.

The results showed no overall significant differences between groups in terms of mean response speed. We also found no significant group differences in the mean accuracy, which was overall very high (above 85% of not anticipated responses). These findings confirmed that the task was equally difficult for participants with DS and TD-MA children. We found that in all participants task performance was implicitly biased by temporal expectancy generated on the basis of local prediction. This was demonstrated by significantly faster (although more inaccurate) responses in trials with long than short preparatory (SOA) intervals. In other words, the longer participants waited for the presentation of the imperative stimulus, the higher was the conditional probability of its onset, hence, the faster they were to detect it. Yet, the downside of this greater motor preparation was a loss of control, with a higher number of anticipation errors for the expected imperative stimuli. The effect of local expectation on behavioral performance is well known and has been consistently reported in typically developing adults (see Los, 2010 for a review) and children (Vallesi and Shallice, 2007; Johnson et al., 2015; Mento and Tarantino, 2015; Mento and Vallesi, 2016). Traditionally, it has been explained by assuming that the probability of an event to occur at a given moment is conditioned by the local accumulation of evidence that has not yet occurred (Niemi and Näätänen, 1981; Luce, 1986). Here we report that task performance was equally impacted by SOA duration in both groups, confirming our recent observation that local prediction is present and operating in the DS (Mento et al., 2019). From a theoretical point of view, our findings also corroborate the hypothesis that the ability to use local prediction (i.e., the foreperiod effect) does not require effortful cognitive processes such as strategic or voluntary control of attention and preparation over time (Van Der Lubbe et al., 2004; Los and Heslenfeld, 2005; Los, 2010; Mento and Tarantino, 2015; Los et al., 2017; Mento et al., 2019).

In spite of preserved speed, accuracy and local prediction performance, we found a remarkable group difference in the ability to extract and use global patterns to generate feedforward motor control. Indeed, in typically developing children, we observed a significant response speeding up to the imperative stimulus in the blocks with a higher frequency of short SOA trials as compared to those with uniform or long-biased distributions. This means that as the task became faster in stimulus presentation, typically developing children were able to adapt their performance, showing faster reaction times (although this implied a slight decrement in response accuracy). Noteworthy, this adaptation did not occur in individuals with DS, who exhibited same accuracy and mean response speed for different global predictive patterns. In other words, although participants with DS were able to implement a basic form of proactive motor control based on the simple accumulation of local expectancy, this ability was not flexibly modulated by global context.

These results provide a possible new avenue for a deeper understanding of the nature of implicit mechanisms in atypical development. In this regard and as far as we know, previous studies claiming that implicit learning is preserved in children with DS (Parkin et al., 1990; Wyatt and Conners, 1998; Vicari et al., 2000; Jones et al., 2002) mainly used task requiring to learn implicitly *vs.* explicitly from a static pattern. Here, for the first time we used a new kind of task defined as Dynamic Temporal Prediction (DTP; Mento and Granziol, under revision) that purposely introduced hierarchically nested, local-global predictive patterns in a dynamic context. The sequence of local (SOA duration) and global (block-type) were fully randomized for each participant, so that they were required to extract consistent rules from a changing sensory stimulation. This methodological aspect allowed us to test not only the *tout-court* presence of implicit learning on motor performance. Rather, it provided a new way to test how flexible and adaptive this cognitive function is. In a recent study using the same task (Mento and Granziol, under revision) we found that the ability to use global patterns to feedforward adapt the motor behavior is already in place from 5 years onward, although it reaches a stable developmental trajectory after 8 years of age. From a developmental perspective the changes in global cognition compared to the stable age trend of local cognition suggests that global cognition generally requires a more developed statistical learning ability, including the capacity to extrapolate and introject even more complex sensory patterns based on hierarchical composite relationships between single elements. This account is in line with Elman’s (Elman, 1993) hypothesis of the “importance of starting small,” (p.72) suggesting that developmental constraints on learning may constitute a necessary prerequisite for mastering complex domains. Also in line with a neuroconstructive theoretical account, a possible way to reconcile previous findings of preserved implicit learning in DS, our findings suggest that perhaps implicit learning, albeit present, is nevertheless less flexible than in typical development. Limited flexibility of implicit mechanisms in the DS may be an early, domain-general constraint whose developmental effect translates into an impairment of explicit cognition, although additional empirical evidence is needed to support this hypothesis.

## Data Availability Statement

The datasets generated for this study are available on request to the corresponding author.

## Ethics Statement

The studies involving human participants were reviewed and approved by Ethics Committee of the School of Psychology at the University of Padua (protocol no 2536). Written informed consent to participate in this study was provided by the participants’ legal guardian/next of kin.

## Author Contributions

GM contributed conception and design of the study. GM and UG organized the database. UG performed the statistical analysis. GM, UG, and GS wrote the first draft of the manuscript. All authors contributed manuscript revision read and approved the submitted version.

## Conflict of Interest

The authors declare that the research was conducted in the absence of any commercial or financial relationships that could be construed as a potential conflict of interest.

## Footnotes

1 Degrees of freedom are calculated accordingly to the kenward-roger approximation (Kenward and Roger, 1997).

## References

[B1] Amso D. Davidow J. (2012). The development of implicit learning from infancy to adulthood: item frequencies, relations, and cognitive flexibility. *Dev. Psychobiol.* 54 664–673. 10.1002/dev.20587 22714674

[B2] Atkinson J. Braddick O. (2011). “From genes to brain development to phenotypic behavior,” in *Progress in Brain Research*, eds Waxman S. Stein D. G. Swaab D. (Amterdem: Elsevier) 261–283. 10.1016/B978-0-444-53884-0.00029-24 21489394

[B3] Bates D. Mächler M. Bolker B. Walker S. (2015). Fitting Linear Mixed-Effects Models Using lme4. *J. Stat. Softw.* 67 1–48. 10.18637/jss.v067.i01

[B4] Belacchi C. Cannoni E. Cornoldi C. (2008). *CPM. Coloured Progressive Matrices. Standarizzazione Italiana.* Firenze: Giunti Organizzazioni Speciale.

[B5] Benjamini Y. Yekutieli D. (2001). The control of the false discovery rate in multiple testing under dependency. *Ann. Stat.* 29 1165–1188.

[B6] Borella E. Carretti B. Lanfranchi S. (2013). Inhibitory mechanisms in Down syndrome: is there a specific or general deficit? *Res. Dev. Disabil.* 34 65–71. 10.1016/j.ridd.2012.07.017 22940160

[B7] Breckenridge K. Braddick O. Anker S. Woodhouse M. Atkinson J. (2013). Attention in Williams syndrome and Down’s syndrome: performance on the new early childhood attention battery. *Br. J. Dev. Psychol.* 31 257–269. 10.1111/bjdp.12003 23659894

[B8] Brenner L. A. (2012). *Temporal Processing and Neurodevelopmental Disorders: Insights from Attention-Deficit/Hyperactivity Disorder, Autism Spectrum Disorder, and 22q11.2 Deletion Syndrome. UCLA.* ProQuest ID: Brenner_ucla_0031D_10462. Merritt ID: ark:/13030/m5t177q4 Available online at: https://escholarship.org/uc/item/2sp4g726.

[B9] Brown J. H. Johnson M. H. Paterson S. J. Gilmore R. Longhi E. Karmiloff-Smith A. (2003). Spatial representation and attention in toddlers with Williams syndrome and Down syndrome. *Neuropsychologia* 41 1037–1046. 10.1016/s0028-3932(02)00299-3 12667539

[B10] Carney D. P. J. J. Brown J. H. Henry L. A. (2013). Executive function in Williams and Down syndromes. *Res. Dev. Disabil.* 34 46–55. 10.1016/j.ridd.2012.07.013 22940158

[B11] Cornish K. Scerif G. Karmiloff-Smith A. (2007). Tracing syndrome-specific trajectories of attention across the lifespan. *Cortex* 43 672–685. 10.1016/s0010-9452(08)70497-0 17710820

[B12] Cornoldi C. Gardinale M. Masi A. Pettenò L. (1996). *Impulsività e Autocontrollo.* Trento: Erikson.

[B13] Correa A. (2010). “Enhancing behavioural performance by visual temporal orienting,” in *Attention and Time*, eds Coull J. Nobre A. (Oxford: Oxford University Press), 359–370. 10.1093/acprof:oso/9780199563456.003.0026

[B14] Correa A. Lupiáñez J. Madrid E. Tudela P. (2006). Temporal attention enhances early visual processing: a review and new evidence from event-related potentials. *Brain Res.* 1076 116–128. 10.1016/j.brainres.2005.11.074 16516173

[B15] Correa A. Lupiáñez J. Milliken B. Tudela P. (2004). Endogenous temporal orienting of attention in detection and discrimination tasks. *Percept. Psychophys* 66 264–278. 10.3758/BF03194878 15129748

[B16] Costanzo F. Varuzza C. Menghini D. Addona F. Gianesini T. Vicari S. (2013). Executive functions in intellectual disabilities: a comparison between Williams syndrome and Down syndrome. *Res. Dev. Disabil.* 34 1770–1780. 10.1016/j.ridd.2013.01.024 23501586

[B17] Coull J. T. (2010). “Neural substrates of temporal attentional orienting - Oxford Scholarship,” in *Attention to Time*, eds Nobre A. Coull J. (Oxford: Oxford University Press).

[B18] Coull J. T. Frith C. D. Büchel C. Nobre A. C. Bu C. Nobre A. C. (2000). Orienting attention in time: behavioural and neuroanatomical distinction between exogenous and endogenous shifts. *Neuropsychologia* 38 808–819. 10.1016/s0028-3932(99)00132-3 10689056

[B19] Daunhauer L. A. Gerlach-McDonald B. Will E. Fidler D. J. (2017). Performance and Ratings Based Measures of Executive Function in School-Aged Children with Down Syndrome. *Dev. Neuropsychol.* 42 351–368. 10.1080/87565641.2017.1360303 28985480

[B20] D’Souza D. Booth R. Connolly M. Happé F. Karmiloff-Smith A. (2016). Rethinking the concepts of ‘local or global processors’: evidence from Williams syndrome. Down syndrome, and Autism Spectrum Disorders. *Dev. Sci.* 19 452–468. 10.1111/desc.12312 26010432 PMC4789488

[B21] Elman J. L. (1993). Learning and development in neural networks: the importance of starting small. *Cognition* 48 71–99. 10.1016/0010-0277(93)90058-4 8403835

[B22] Fox J. Weisberg S. (2018). *An R companion to Applied Regression.* Thousand Oaks, MA: SAGE Publications.

[B23] Johnson K. A. Burrowes E. Coull J. T. (2015). Children can implicitly, but not voluntarily, direct attention in time. *PLoS One* 10:e0123625. 10.1371/journal.pone.0123625 25881188 PMC4399911

[B24] Johnson S. Fernandes K. Frank M. Kirkham N. Marcus G. Rabagliati H. (2009). Abstract Rule Learning for Visual Sequences in 8- and 11-Month-Olds. *Infancy* 14 2–18. 10.1080/15250000802569611 19283080 PMC2654175

[B25] Jones R. S. P. Vaughan F. L. Roberts M. (2002). Mental retardation and memory for spatial locations. *Am. J. Ment. Retard.* 107 99–104. 10.1352/0895-80172002107<0099:MRAMFS<2.0.CO;2 11853527

[B26] Karlin L. (1958). Reaction time as a function of foreperiod duration and variability. *J. Exp. Psychol. Gen.* 58 185–191. 10.1037/h0049152 14404508

[B27] Karmiloff-Smith A. (1998). Development itself is the key to understanding developmental disorders. *Trends Cogn. Sci.* 2 389–398. 10.1016/s1364-6613(98)01230-3 21227254

[B28] Kenward M. G. Roger J. H. (1997). Small Sample Inference for Fixed Effects from Restricted Maximum Likelihood. *Biometrics* 53:983 10.2307/2533558 9333350

[B29] Lanfranchi S. Cornoldi C. Vianello R. (2004). Verbal and visuospatial working memory deficits in children with Down syndrome. *Am. J. Ment. Retard.* 109 456–466. 10.1352/0895-80172004109<456:VAVWMD<2.0.CO;2 15471512

[B30] Lanfranchi S. Jerman O. Dal Pont E. Alberti A. Vianello R. (2010). Executive function in adolescents with Down Syndrome. *J. Intellect. Disabil. Res.* 54 308–319. 10.1111/j.1365-2788.2010.01262.x 20202074

[B31] Lee N. R. Fidler D. J. Blakeley-Smith A. Daunhauer L. Robinson C. Hepburn S. L. (2011). Caregiver report of executive functioning in a population-based sample of young children with Down syndrome. *Am. J. Intellect. Dev. Disabil.* 116 290–304. 10.1352/1944-7558-116.4.290 21740257 PMC4512645

[B32] Lenth R. (2018). *Emmeans: Estomated Marginal Means, Aka Least Square Means. R Package Version, 1(2).*

[B33] Los S. A. (2010). “Foreperiod and sequential effects: theory and data,” in *Attention and Time*, eds Coull J. Nobre A. C. (Oxford: Oxford University Press), 289–302. 10.1093/acprof:oso/9780199563456.003.0021

[B34] Los S. A. Heslenfeld D. J. (2005). Intentional and unintentional contributions to nonspecific preparation: electrophysiological evidence. *J. Exp. Psychol. Gen.* 134 52–72. 10.1037/0096-3445.134.1.52 15702963

[B35] Los S. A. Kruijne W. Meeter M. (2017). Hazard versus history: temporal preparation is driven by past experience. *J. Exp. Psychol. Hum. Percept. Perform.* 43 78–88. 10.1037/xhp0000279 27808547

[B36] Luce R. D. (1986). *Response Times: Their Role in Inferring Elementary Mental Organization.* Oxford: OUP USA.

[B37] McGrother C. W. Marshall B. (2008). Recent trends in incidence, morbidity and survival in Down’s syndrome. *J. Intellect. Disabil. Res.* 34 49–57. 10.1111/j.1365-2788.1990.tb01514.x 2139131

[B38] Mento G. (2017). The role of the P3 and CNV components in voluntary and automatic temporal orienting: a high spatial-resolution ERP study. *Neuropsychologia* 107 31–40. 10.1016/j.neuropsychologia.2017.10.037 29109036

[B39] Mento G. Astle D. E. Scerif G. (2018). Cross-frequency phase–amplitude coupling as a mechanism for temporal orienting of attention in childhood. *J. Cogn. Neurosci.* 30 594–602. 10.1162/jocn_a_01223 29244640

[B40] Mento G. Scerif G. Granziol U. Franzoi M. Lanfranchi S. (2019). Dissociating top-down and bottom-up temporal attention in Down syndrome: a neurocostructive perspective. *Cogn. Dev.* 49 81–93. 10.1016/J.COGDEV.2018.12.004

[B41] Mento G. Tarantino V. (2015). Developmental trajectories of internally and externally driven temporal prediction. *PLoS One* 10:e0135098. 10.1371/journal.pone.0135098 26262878 PMC4532408

[B42] Mento G. Tarantino V. Vallesi A. Bisiacchi P. S. P. S. (2015). Spatiotemporal neurodynamics underlying internally and externally driven temporal prediction: a high spatial resolution ERP study. *J. Cogn. Neurosci.* 27 425–439. 10.1162/jocn_a_00715 25203276

[B43] Mento G. Vallesi A. (2016). Spatiotemporally dissociable neural signatures for generating and updating expectation over time in children: a high density-ERP study. *Dev. Cogn. Neurosci.* 19 98–106. 10.1016/j.dcn.2016.02.008 26946428 PMC6988099

[B44] Meulemans T. Van der Linden M. Perruchet P. (1998). Implicit Sequence Learning in Children. *J. Exp. Child Psychol.* 69 199–221. 10.1006/jecp.1998.2442 9654439

[B45] Niemi P. Näätänen R. (1981). Foreperiod and simple reaction time. *Psychol. Bull.* 89 133–162. 10.1037/0033-2909.89.1.133

[B46] Nobre A. Correa A. Coull J. (2007). The hazards of time. *Curr. Opin. Neurobiol.* 17 465–470. 10.1016/j.conb.2007.07.006 17709239

[B47] Nobre A. C. (2001). Orienting attention to instants in time. *Neuropsychologia* 39 1317–1328. 10.1016/s0028-3932(01)00120-8 11566314

[B48] Parkin A. J. Reid T. K. Russo R. (1990). On the differential nature of implicit and explicit memory. *Mem. Cogn.* 18 507–514. 10.3758/BF03198483 2233263

[B49] R Core Team (2018). *R: A Language and Environment for Statistical Computing.* Vienna: R Foundation for Statistical Computing.

[B50] Raven J. C. Court J. H. Raven J. (1990). *Manual for Raven’s Progressive Matrices and Vocabulary Scales: Coloured Progressive Matrices.* London: Oxford University Press.

[B51] Scerif G. Steele A. (2011). Neurocognitive development of attention across genetic syndromes: inspecting a disorder’s dynamics through the lens of another. *Prog. Brain Res.* 189 285–301. 10.1016/B978-0-444-53884-0.00030-30 21489395

[B52] Trillenberg P. Verleger R. Wascher E. Wauschkuhn B. Wessel K. (2000). CNV and temporal uncertainty with ‘ageing’ and ‘non-ageing’. *Clin. Neurophysiol.* 111 1216–1226. 10.1016/s1388-2457(00)00274-1 10880797

[B53] Vallesi A. (2010). “Neuro-anatomical substrates of foreperiod effects,” in *Attention and Time*, eds Coull J. T. Nobre A. C. (Oxford: Oxford University Press), 303–316. 10.1093/acprof:oso/9780199563456.003.0022

[B54] Vallesi A. Shallice T. (2007). Developmental dissociations of preparation over time: deconstructing the variable foreperiod phenomena. *J. Exp. Psychol. Hum. Percept. Perform.* 33 1377–1388. 10.1037/0096-1523.33.6.1377 18085950

[B55] Van Der Lubbe R. H. J. Los S. A. Ja P. (2004). Being prepared on time: on the importance of the previous foreperiod to current preparation, as reflected in speed, force and preparation-related brain potentials. *Acta Psychol.* 116 245–262. 10.1016/j.actpsy.2004.03.003 15222969

[B56] Vicari S. Bellucci S. Carlesimo G. A. (2000). Implicit and explicit memory: a functional dissociation in persons with Down syndrome. *Neuropsychologia* 38 240–251. 10.1016/s0028-3932(99)00081-0 10678691

[B57] Vinter A. Perruchet P. (2010). Implicit learning in children is not related to age: evidence from drawing behavior. *Child Dev.* 71 1223–1240. 10.1111/1467-8624.00225 11108093

[B58] Westfall J. Kenny D. A. Judd C. M. (2014). Statistical power and optimal design in experiments in which samples of participants respond to samples of stimuli. *J. Exp. Psychol. Gen.* 143 2020–2045. 10.1037/xge0000014 25111580

[B59] Woodrow H. (1914). The measurement of attention. *Psychol. Monogr.* 17 1–158.

[B60] Wyatt B. S. Conners F. A. (1998). Implicit and explicit memory in individuals with mental retardation. *Am. J. Ment. Retard.* 102 511–526. 9544347 10.1352/0895-8017(1998)102<0511:iaemii>2.0.co;2

